# Comparative safety outcomes of three venous access routes for totally implantable venous access port placement and exploratory assessment of pre-removal catheter-related thrombosis in breast cancer patients

**DOI:** 10.3389/fonc.2026.1934294

**Published:** 2026-09-30

**Authors:** Mintao Li, Chao Ma, Feng Lu, Yubin He, Kangli Yin, Jialong Li, Yuanyuan Xu, Jian Dong

**Affiliations:** Department of Vascular Surgery, Shanghai Traditional Chinese Medicine Integrated Hospital, Shanghai, China

**Keywords:** breast neoplasms, catheter-related thrombosis, d-dimer, implantation route, risk assessment, totally implantable venous access port

## Abstract

**Objective:**

To compare the safety outcomes of totally implantable venous access port (TIVAP) implantation through the internal jugular vein (IJV), subclavian vein (SCV), and axillary vein (AxV) in patients with breast cancer, and to explore a pre-removal thrombosis risk-stratification framework integrating D-dimer levels and vascular ultrasonography findings.

**Methods:**

Data from 475 breast cancer patients who underwent TIVAP implantation were retrospectively reviewed. Patients were classified according to implantation route: IJV (n = 161), SCV (n = 138), and AxV (n = 176). Baseline characteristics, procedure variables, postoperative complications, and Clavien–Dindo grades were analyzed. Among these patients, 234 underwent both pre-removal D-dimer testing and vascular ultrasonography, and 18 asymptomatic thrombotic events were identified. Firth penalized logistic regression was used to explore factors associated with thrombosis. ROC curve analysis was performed to evaluate the discriminatory ability of D-dimer, and the optimal cutoff was determined using the Youden index. Patients were categorized into four scenarios according to D-dimer and ultrasonography findings.

**Results:**

Baseline characteristics were comparable among the three groups. The complication rate was higher in the SCV group than in the IJV and AxV groups (16.67% vs. 7.45% vs. 5.68%, P = 0.002). Catheter fracture and pinch-off syndrome occurred exclusively in patients receiving SCV access. Firth penalized logistic regression showed that higher D-dimer levels and longer catheter dwell time were associated with asymptomatic catheter-related thrombosis. D-dimer showed moderate discrimination (AUC = 0.721). At the ROC-derived cutoff of 0.251 μg/mL, sensitivity was 77.78%, specificity was 76.85%, LR+ was 3.36, and LR− was 0.29. Integration of D-dimer and ultrasonography yielded four exploratory pre-removal profiles, including 166 double-negative, 4 D-dimer-negative/ultrasound-positive, 50 D-dimer-positive/ultrasound-negative, and 14 double-positive patients.

**Conclusion:**

Compared with the SCV approach, the AxV approach showed a lower observed complication rate in this retrospective cohort, whereas no statistically significant difference in overall complications was observed between the AxV and IJV groups. D-dimer alone was insufficient to exclude thrombosis. The combined D-dimer/ultrasonography framework should be regarded as exploratory and requires prospective validation before its potential clinical application can be considered.

## 1 Introduction

The totally implantable venous access port (TIVAP) is a fully implanted central venous access device widely used in patients requiring long-term intravenous therapy, particularly those undergoing chemotherapy, parenteral nutrition, or repeated blood product administration (1). Compared with conventional central venous catheters, TIVAPs provide several advantages, including a lower risk of infection, improved patient comfort, and longer intervals between maintenance procedures (2). With the increasing use of long-term intravenous treatment in oncology patients, optimizing implantation techniques and minimizing device-related complications have become important considerations in clinical practice (3).

Several venous access routes have been described for chest wall TIVAP implantation, among which the internal jugular vein (IJV), subclavian vein (SCV), and axillary vein (AxV) are the most commonly used. The IJV and SCV approaches remain the traditional access routes. Ultrasound-guided IJV puncture has been recommended by several guidelines as a preferred approach because of its relatively predictable anatomy and lower risk of procedure-related complications, particularly pneumothorax (4). However, the optimal access route remains a matter of debate. Previous studies have suggested that the SCV approach is associated with higher rates of overall complications, catheter malposition, and bloodstream infection compared with the IJV approach (5–7), whereas other investigations have reported comparable complication rates among different implantation routes (8).

A major concern specific to the SCV approach is pinch-off syndrome (POS). Initially described by Hinke et al. (9), POS results from repetitive compression of the catheter between the clavicle and the first rib, potentially causing catheter stenosis, fracture, or even embolization of catheter fragments. Because of these mechanical limitations, alternative access routes such as the AxV have attracted increasing attention (10). Anatomically, the AxV is located lateral to the costoclavicular compression area, theoretically reducing the risk of catheter compression-related complications (11). Previous retrospective studies have demonstrated favorable outcomes with ultrasound-guided AxV access, including low overall complication rates and a low incidence of catheter-related thrombosis (12, 13). In a randomized controlled trial, Chen et al. (14) reported that the AxV/SCV approach provided improved patient comfort and shorter procedural times compared with the IJV approach, although a higher incidence of early complications, particularly catheter malposition, was observed.

Catheter-related thrombosis (CRT) remains one of the most frequent complications associated with TIVAP placement, and a substantial proportion of thrombotic events are asymptomatic and detected only through imaging examination (15). D-dimer is widely used as a biomarker for thrombotic screening; however, its clinical value in predicting catheter-associated thrombosis remains uncertain. Liu et al. (16) reported that D-dimer had limited value in excluding PICC-related thrombosis, suggesting that a normal D-dimer level may not reliably rule out thrombus formation. In contrast, other studies have shown that elevated D-dimer levels are associated with increased thrombotic risk and that dynamic changes in D-dimer may provide predictive information for catheter-related thrombotic events (17). Nevertheless, whether pre-removal D-dimer measurement can identify asymptomatic mural thrombosis before TIVAP removal has not been adequately investigated.

Several unresolved issues remain. First, comparative studies evaluating long-term complications among the IJV, SCV, and AxV approaches under standardized implantation protocols are still limited. Second, the diagnostic performance and optimal threshold of pre-removal D-dimer for detecting asymptomatic TIVAP-associated thrombosis have not been clearly established. Third, evidence-based approaches integrating laboratory markers and vascular imaging for pre-removal thrombotic risk characterization remain limited.

Therefore, this study evaluated the safety outcomes of three TIVAP implantation routes (IJV, SCV, and AxV) in breast cancer patients and investigated the association between pre-removal D-dimer levels and ultrasonographically detected thrombosis. In addition, a four-scenario exploratory risk-stratification framework combining D-dimer results and vascular ultrasonography findings was explored to characterize heterogeneous pre-removal thrombotic risk profiles. Given the retrospective design and the limited number of thrombotic events, this exploratory framework was intended to generate hypotheses for future prospective validation rather than establish a validated clinical decision tool.

## 2 Materials and methods

### 2.1 Study population

This retrospective study reviewed the clinical data of breast cancer patients who underwent TIVAP implantation and subsequent port removal at the Department of Vascular Surgery, Shanghai Integrated Traditional Chinese and Western Medicine Hospital, between January 2022 and December 2025. For catheter-related outcomes, patients were observed during the period in which the TIVAP remained *in situ*, from implantation until port removal or the last available clinical follow-up. The duration of catheter exposure was recorded as catheter dwell time. The pre-removal thrombosis analysis specifically evaluated ultrasound-detected thrombosis at the scheduled port-removal assessment.

Patients were eligible if they met the following inclusion criteria: (1) female patients with pathologically confirmed breast cancer who received chemotherapy; (2) TIVAP implantation performed through the internal jugular vein, subclavian vein, or axillary vein; (3) age ≥18 years; and (4) complete clinical and follow-up records available. Patients were excluded if they had: (1) pre-existing central venous thrombosis; (2) known coagulation disorders; (3) severe hepatic or renal dysfunction; or (4) incomplete clinical data or loss to follow-up.

A total of 475 patients were included and categorized according to the implantation route: the IJV group (n = 161), SCV group (n = 138), and AxV group (n = 176). Among the 475 patients who underwent TIVAP implantation, 234 patients completed their planned anticancer treatment during the study period and subsequently underwent port-removal evaluation. These patients received pre-removal D-dimer testing and vascular ultrasonography according to the institutional clinical workflow and were included in the analysis of asymptomatic catheter-related thrombosis. The remaining 241 patients had not reached the port-removal stage because they continued to require venous access for ongoing treatment or follow-up and were therefore not included in the pre-removal thrombosis analysis. The study protocol was approved by the IRB of Shanghai TCM-Integrated Hospital (Approval No. 2026-025-1). The requirement for informed consent was waived because of the retrospective nature of the study.

### 2.2 Surgical procedure

All TIVAP implantation procedures were performed under local anesthesia by two experienced vascular surgeons using a power-injectable implantable port system and compatible accessories (PowerPort 8806061, BARD, USA).

Patients were placed in the supine position with elevation of the ipsilateral shoulder and rotation of the head toward the contralateral side. Venous puncture and catheter advancement were performed under continuous real-time ultrasound guidance. The catheter tip position was confirmed by intraoperative fluoroscopy and adjusted to the junction of the lower third of the superior vena cava and the right atrium. Representative postoperative chest radiographs demonstrating catheter placement through the three access routes are shown in **Figure 1**.

**Figure 1:**
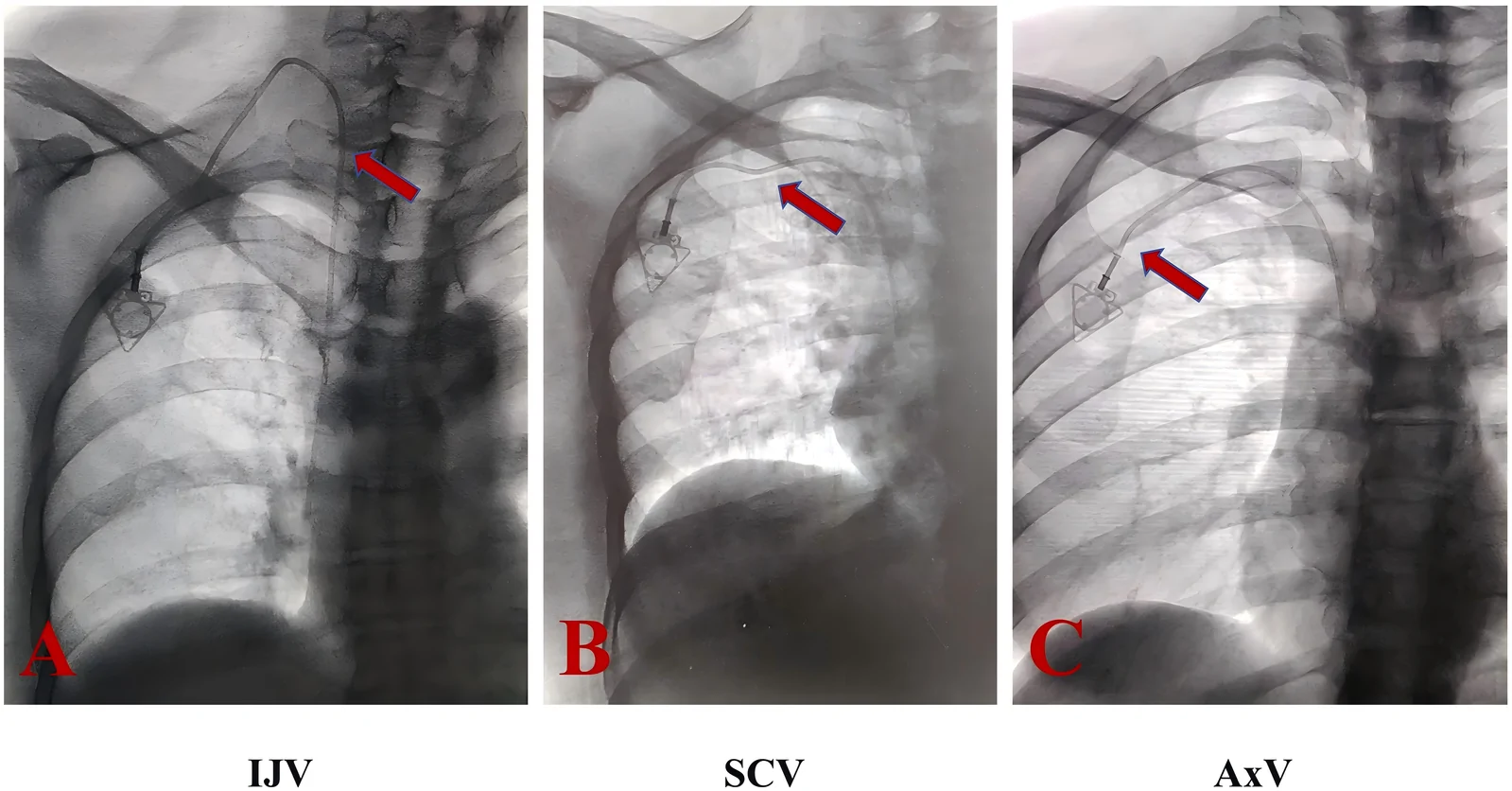
Representative radiographic images of TIVAP implantation through three venous access routes. **(A)** TIVAP implantation through the internal jugular vein (IJV) approach. **(B)** TIVAP implantation through the subclavian vein (SCV) approach. **(C)** TIVAP implantation through the axillary vein (AxV) approach.

IJV group: The internal jugular vein was punctured under ultrasound guidance using the modified Seldinger technique at the junction between the sternal and clavicular heads of the sternocleidomastoid muscle. After guidewire placement and fluoroscopic confirmation, the catheter sheath was advanced. The catheter was then tunneled subcutaneously to the chest wall pocket, and the port reservoir was secured to the deep fascia using non-absorbable sutures. SCV group:The subclavian vein was punctured inferior to the junction of the medial and middle thirds of the clavicle. A transverse skin incision approximately 2–3 cm in length was made along the guidewire trajectory, followed by blunt dissection of subcutaneous tissue to create the port pocket. AxV group:The third segment of the axillary vein was punctured under ultrasound guidance. Subsequent catheter advancement and port implantation procedures were performed using the same technique as that used in the SCV group.

### 2.3 Outcome measures and data collection

Clinical data were collected from electronic medical records, laboratory databases, and vascular ultrasound imaging systems.

The following variables were analyzed: (1) Baseline characteristics: age, body mass index (BMI), tumor stage, chemotherapy regimen (single-agent or combination therapy), and catheterization side; (2) Procedure-related parameters: implantation route, operative time, and intraoperative complications; (3) Catheter-related information: implantation date, removal date, and total catheter dwell time; (4) Laboratory parameters: pre-removal D-dimer level, platelet count, prothrombin time (PT), activated partial thromboplastin time (APTT), and fibrinogen level; (5) Vascular ultrasonography findings: presence or absence of thrombus, thrombus location, and thrombus characteristics (mural or occlusive); Pre-removal venous ultrasonography was performed using high-resolution ultrasound with compression, color Doppler, and spectral Doppler techniques. The examination included the catheter-related venous segments, including the internal jugular vein, subclavian/axillary vein, and other accessible central venous segments according to anatomical feasibility. Catheter-related thrombosis was defined as the presence of non-compressibility, intraluminal echogenic material, abnormal Doppler flow, or complete/partial occlusion associated with the catheter. Ultrasound examinations were performed by experienced vascular sonographers. Ultrasonographers were blinded to the corresponding D-dimer results at the time of image acquisition and interpretation. (6) Postoperative complications: catheter-related infection, catheter occlusion, catheter rupture, pinch-off syndrome, pneumothorax, arrhythmia, and poor wound healing. Complication severity was graded according to the Clavien–Dindo classification system (grades I–V). For the pre-removal thrombosis analysis, the primary thrombotic endpoint was asymptomatic catheter-related thrombosis, defined as ultrasound-detected thrombus in the absence of corresponding clinical symptoms at the time of scheduled port removal. D-dimer testing and vascular ultrasonography were performed within 48 hours before scheduled port removal. The interval between blood sampling and ultrasound examination did not exceed 24 hours.

### 2.4 Grouping and definitions

#### 2.4.1 Route comparison analysis

The 475 patients who underwent TIVAP implantation were divided into three groups according to the implantation route: IJV, SCV, and AxV groups.

#### 2.4.2 Thrombosis risk analysis

Among the 234 patients undergoing port removal, patients were classified into the thrombosis group or non-thrombosis group according to the presence or absence of ultrasound-detected thrombus.

#### 2.4.3 Four-scenario exploratory framework

Patients undergoing port removal were further categorized according to the combination of pre-removal D-dimer levels and vascular ultrasonography findings. For descriptive comparisons in **Table 1**, elevated D-dimer was defined according to the institutional laboratory upper reference limit of 0.243 μg/mL. For the exploratory four-scenario framework, the ROC-derived cutoff of 0.251 μg/mL was used. Scenario A: D-dimer <0.251 μg/mL and no ultrasound-detected thrombosis; Scenario B: D-dimer <0.251 μg/mL with ultrasound-detected thrombosis;

**Table 1:** Comparison of clinical and laboratory parameters between thrombosis and non-thrombosis groups in port-removal patients (n = 234).

Variable	Thrombosis group (n=18)	Non-thrombosis group (n=216)	*P value*
Laboratory parameters			
D-dimer (μg/mL, median, Q1-Q3)	0.361(0.190,1.290)	0.019(0.010,0.240)	0.002
D-dimer category, n (%)			
Elevated D-dimer	15 (83.3)	52 (24.1)	<0.001
Normal D-dimer	3 (16.7)	164 (75.9)	
Platelet count (×10^9^/L), mean ± SD	252.3 ± 54.1	246.7 ± 52.8	0.670
Fibrinogen (g/L), mean ± SD	3.62 ± 0.65	3.42 ± 0.62	0.180
Clinical characteristics			
Age (years), mean ± SD	51.8 ± 6.9	52.1 ± 8.2	0.890
Catheter dwell time (months), median (Q1–Q3)	10.65 (8.10–14.00)	8.65 (6.30–10.80)	0.010
Original implantation route, n (%)			0.251
IJV	6 (33.3)	73 (33.8)	
SCV	8 (44.4)	60 (27.8)	
AxV	4 (22.2)	83 (38.4)	

The 0.243 μg/mL threshold represents the institutional laboratory upper reference limit and was used for descriptive group comparison; the ROC-derived cutoff of 0.251 μg/mL was used for the four-scenario framework.

Scenario C: D-dimer ≥0.251 μg/mL without ultrasound-detected thrombosis; Scenario D: D-dimer ≥0.251 μg/mL with ultrasound-detected thrombosis.

#### 2.4.4 Institutional anticoagulation management patterns during the study period

Anticoagulation management was not prospectively assigned according to the exploratory four-scenario framework but was determined according to institutional practice and individual clinical assessment during the study period. For patients with ultrasound-confirmed catheter-related thrombosis identified before port removal, anticoagulation therapy was initiated based on thrombotic and bleeding risk evaluation. During the study period, low-molecular-weight heparin (LMWH, 100 IU/kg subcutaneously every 12 hours) or rivaroxaban was used according to clinical preference. Port removal timing was determined individually after assessment of thrombus status and treatment response. For patients with elevated D-dimer levels without ultrasound-confirmed thrombosis, management was individualized after assessment of possible causes of D-dimer elevation and thrombotic/bleeding risks. Some patients received short-term anticoagulation according to institutional practice; however, this retrospective study was not designed to determine whether anticoagulation provides clinical benefit in patients with isolated D-dimer elevation. For patients receiving anticoagulation before port removal, repeat ultrasonography was performed when clinically indicated to reassess thrombus status. The observed reassessment intervals represented institutional practice patterns in this retrospective cohort and should not be interpreted as recommended anticoagulation durations. The complete post-removal anticoagulation course was not systematically captured in the dataset.

### 2.5 Statistical analysis

Statistical analyses were performed using SPSS version 29.0 and R version 4.5.3. Normality of continuous variables was assessed using the Shapiro–Wilk test. Normally distributed variables are presented as mean ± SD; comparisons among the three implantation-route groups were performed using one-way ANOVA, whereas *post-hoc* comparisons were performed using the Tukey method. Non-normally distributed variables are presented as median (IQR) and were compared using the Kruskal–Wallis test or Mann–Whitney U test, as appropriate. Categorical variables are presented as n/N (%) or n (%) and were compared using Pearson’s chi-square test or Fisher’s exact test. When the overall comparison among the three implantation groups was statistically significant, pairwise comparisons were performed using Pearson’s chi-square tests with Bonferroni adjustment for three pairwise comparisons. Because only 18 thrombotic events occurred among 234 patients, Firth penalized logistic regression was used to reduce small-sample bias. To minimize overfitting, the primary multivariable model was restricted to three clinically prespecified covariates: D-dimer level, catheter dwell time, and fibrinogen level. These variables were selected on the basis of their clinical relevance and biological plausibility as indicators of thrombotic activity, duration of catheter exposure, and coagulation status, respectively, rather than solely according to univariate statistical significance. ORs and 95% CIs were estimated using Firth penalized likelihood. Bootstrap resampling with 632 iterations was performed as a sensitivity analysis to assess the stability of the associations estimated by the Firth penalized logistic regression model. ROC analysis was performed to evaluate D-dimer discrimination for ultrasound-detected thrombosis. AUC with 95% CI was reported, and the optimal cutoff was determined using the Youden index. Sensitivity, specificity, PPV, NPV, LR+, and LR− were calculated. All tests were two-sided, with P <0.05 considered statistically significant.

## 3 Results

### 3.1 Baseline characteristics

A total of 475 breast cancer patients who underwent TIVAP implantation were included in this study, including 161 patients in the IJV group, 138 in the SCV group, and 176 in the AxV group. Baseline characteristics are summarized in **Table 2**. No significant differences were observed among the three groups regarding age, BMI, tumor stage, chemotherapy regimen, catheterization side, catheter dwell time, operative time, history of ipsilateral breast surgery, or history of ipsilateral radiotherapy (all P > 0.05). No statistically significant differences were detected in the measured baseline characteristics among the three access-route groups. Compared with the overall cohort, patients included in the thrombosis analysis were those who underwent standardized pre-removal assessment. Because this assessment was performed according to institutional practice during the study period, selection bias cannot be completely excluded.

**Table 2:** Baseline characteristics and clinical data of breast cancer patients among the three implantation groups (n = 475).

Variable	IJV (n=161)	SCV (n=138)	AxV (n=176)	*P value*
Demographic data				
Age (years)	52.01 ± 6.81	52.72 ± 7.42	51.6 ± 6.66	0.366
BMI (kg/m²)	22.09 ± 2.50	22.13 ± 2.51	22.14 ± 2.48	0.978
Tumor stage, n/N (%)				
Early (stage I–II)	118/161 (73.3%)	102/138 (73.9%)	129/176 (73.3%)	0.990
Advanced (stage III–IV)	43/161 (26.7%)	36/138 (26.1%)	47/176 (26.7%)	
Chemotherapy regimen, n/N (%)				
Single-agent chemotherapy	35/161 (21.7%)	30/138 (21.7%)	38/176 (21.6%)	0.999
Combination chemotherapy	126/161 (78.3%)	108/138 (78.3%)	138/176 (78.4%)	
Operative data				
Catheterization side, n/N (%)				
Right	97/161 (60.2%)	83/138 (60.1%)	106/176 (60.2%)	1.000
Left	64/161 (39.8%)	55/138 (39.9%)	70/176 (39.8%)	
Catheter dwell time (months)	7.5 (4.72–10.5)	7.8 (5.5–11.5)	8.5 (5.5–12.5)	0.271
Operative time (minutes)	30.06 ± 5.42	31.00 ± 4.91	30.50 ± 5.58	0.351
Prior ipsilateral surgery, n/N (%)				
No	96/161 (59.6%)	85/138 (61.6%)	105/176 (59.7%)	0.925
Yes	65/161 (40.4%)	53/138 (38.4%)	71/176 (40.3%)	
Prior ipsilateral radiotherapy, n/N (%)				
No	120/161 (74.5%)	103/138 (74.6%)	131/176 (74.4%)	0.999
Yes	41/161 (25.5%)	35/138 (25.4%)	45/176 (25.6%)	

Categorical variables are presented as n/N (%), using the number of patients within each implantation-route group as the denominator.

### 3.2 Comparison of complication rates and Clavien–Dindo grades among the three routes

The postoperative complication profiles of the three implantation routes are summarized in **Table 3**. The overall complication rates were 7.45% in the IJV group, 16.67% in the SCV group, and 5.68% in the AxV group, with a significant difference among the three groups (P = 0.002). After Bonferroni adjustment for three pairwise comparisons, the SCV group had a higher overall complication rate than both the IJV group (adjusted P = 0.039) and the AxV group (adjusted P = 0.006), whereas no significant difference was observed between the IJV and AxV groups (adjusted P = 1.000). Among individual complications, catheter fracture and pinch-off syndrome occurred exclusively in the SCV group, with significant differences among the three groups (P = 0.007 and P < 0.001, respectively). AxV was associated with the lowest observed overall complication rate among the three access routes in this cohort. However, these findings should be interpreted cautiously because implantation route selection was not randomized. No significant differences were identified among the three groups in the incidence of pneumothorax, catheter-related infection, arrhythmia, poor wound healing/hematoma, or other complications (all P > 0.05). A forest plot illustrating unadjusted odds ratios of postoperative complications among the three implantation routes, with the IJV group as the reference, is presented in **Figure 2**.

**Table 3:** Complication rates and Clavien–Dindo grades among the three implantation route groups (n=475).

Variable	IJV (n=161)	SCV (n=138)	AxV (n=176)	*P*
Overall complications, n/N (%)	12/161 (7.45%)	23/138 (16.67%)	10/176 (5.68%)	0.002
Clavien-Dindo grade, n/N (%)				0.732
Grade 0 (no complication)	149/161 (92.5%)	115/138 (83.3%)	166/176 (94.3%)	
Grade I (no drug/surgical intervention)	2/161 (1.2%)	2/138 (1.4%)	3/176 (1.7%)	
Grade II (requiring pharmacological treatment)	8/161 (5.0%)	7/138 (5.1%)	6/176 (3.4%)	
Grade IIIa (intervention without general anesthesia)	2/161 (1.2%)	14/138 (10.1%)	1/176 (0.6%)	
Specific complications, n/N (%)				
Catheter-related infection	2/161 (1.2%)	1/138 (0.7%)	1/176 (0.6%)	0.783
Catheter occlusion/thrombosis	3/161 (1.9%)	6/138 (4.3%)	1/176 (0.6%)	0.066
Catheter rupture/fracture	0/161 (0%)	4/138 (2.9%)	0/176 (0%)	0.007
Pinch-off syndrome	0/161 (0%)	8/138 (5.8%)	0/176 (0%)	<0.001
Pneumothorax	0/161 (0%)	1/138 (0.7%)	1/176 (0.6%)	0.339
Arrhythmia	3/161 (1.9%)	2/138 (1.4%)	2/176 (1.1%)	0.858
Poor wound healing/hematoma	4/161 (2.5%)	2/138 (1.4%)	2/176 (1.1%)	0.610
Others (catheter malposition, etc.)	4/161 (2.5%)	5/138 (3.6%)	3/176 (1.7%)	0.560

Patients could experience more than one specific complication; therefore, the sum of individual complications may exceed the total number of patients with complications.

**Figure 2:**
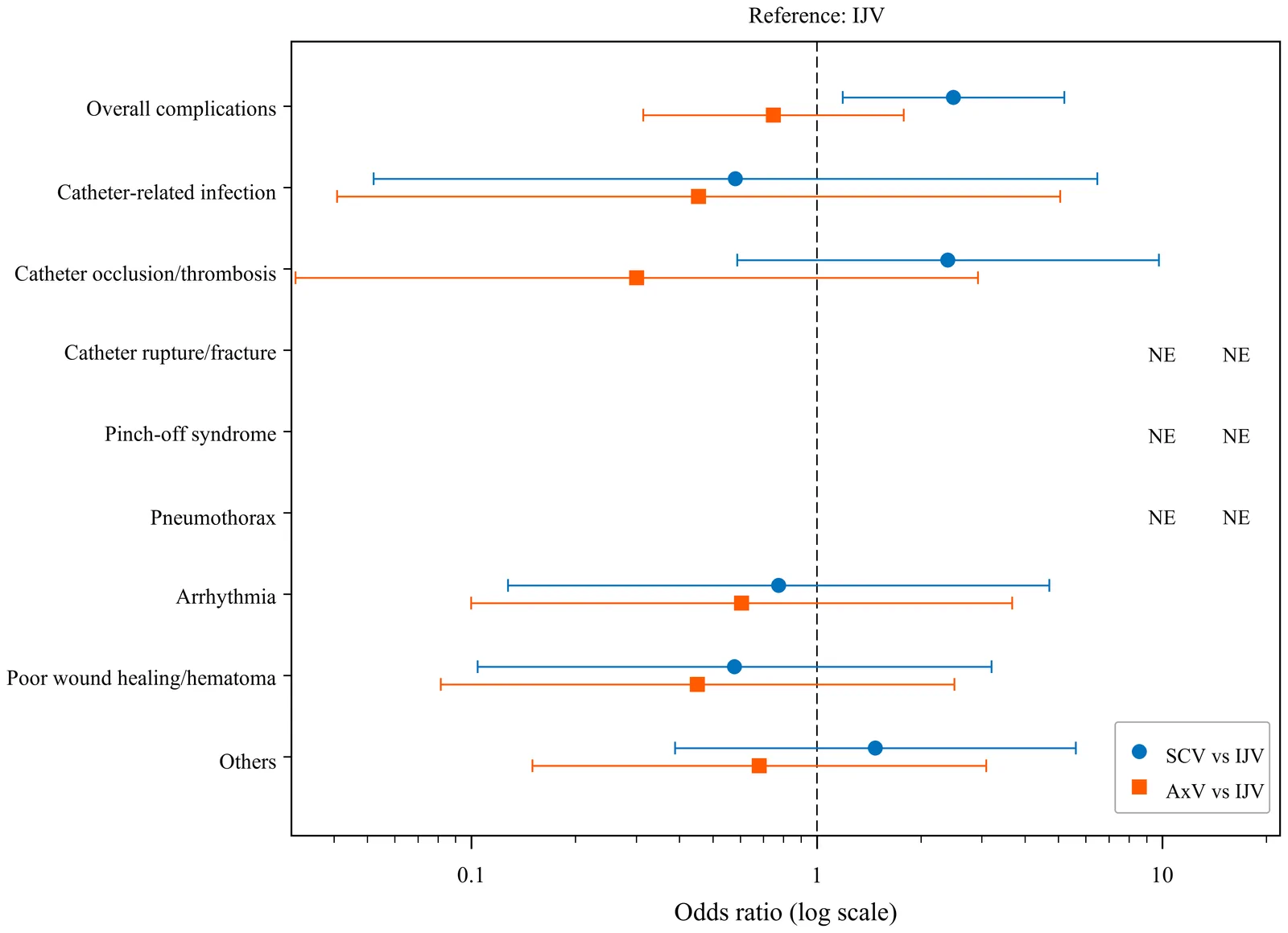
Unadjusted odds ratios for postoperative complications according to TIVAP implantation route. Unadjusted odds ratios (ORs) with 95% confidence intervals (CIs) for postoperative complications were calculated using the internal jugular vein (IJV) group as the reference category. Separate comparisons are presented for the subclavian vein (SCV) versus IJV and axillary vein (AxV) versus IJV groups. ORs are displayed on a logarithmic scale, with OR>1 indicating a risk factor. NE, not estimable; ORs were not calculated when zero events in the reference or comparison group resulted in an undefined estimate or complete separation.

### 3.3 Comparison of clinical and laboratory parameters between thrombosis and non-thrombosis groups in port-removal patients

Among the 234 patients who underwent port removal, vascular ultrasonography identified asymptomatic thrombosis in 18 patients (7.69%). Mural thrombosis was the predominant thrombus type, and the subclavian vein was the most frequently involved site. Clinical and laboratory characteristics of patients with and without thrombosis are compared in **Table 1**. Patients with thrombosis had significantly higher D-dimer levels than those without thrombosis (P = 0.002). Using the institutional laboratory upper reference limit of 0.243 μg/mL, elevated D-dimer levels were observed in 15/18 (83.3%) thrombosis cases and 52/216 (24.1%) non-thrombosis cases (P<0.001). Patients with thrombosis had longer catheter dwell times than those without thrombosis (P = 0.010). No statistically significant differences were observed in platelet count, fibrinogen level, or age between the two groups (P = 0.670, P = 0.180, and P = 0.890, respectively). No significant differences were observed in prothrombin time or activated partial thromboplastin time between the two groups. The rate of thrombus detection did not differ significantly according to the original implantation route among patients undergoing port removal (P = 0.251).

### 3.4 Firth penalized logistic regression analysis of factors associated with catheter-related thrombosis

Because only 18 thrombotic events were identified among the 234 patients undergoing pre-removal assessment, Firth penalized logistic regression was applied to reduce small-sample bias. The analysis showed that elevated D-dimer levels and longer catheter dwell time were associated with catheter-related thrombosis. The corresponding odds ratios and 95% confidence intervals are presented in **Table 4**.

**Table 4:** Firth penalized logistic regression analysis of factors associated with asymptomatic TIVAP-associated thrombosis before port removal (n=234, events=18).

Variable	Univariate analysis	p	Multivariable analysis	*p*
	OR (95% CI)		OR (95% CI)	
D-dimer (per 1 μg/mL increase)	7.254 (2.792,18.850)	<0.001	7.495 (2.280 ~ 24.635)	0.001
Dwell time (per 30-day increase)	1.864 (1.316,2.641)	<0.001	1.623 (1.245 ~ 2.114)	<0.001
Fibrinogen (per 1 g/L increase)	3.602 (1.203,10.780)	0.022	3.250 (0.892 ~ 11.839)	0.074
Platelet count (per 10×10^9^/L increase)	1.035 (0.999,1.073)	0.057		
Age (per 10-year increase)	0.910 (0.818,1.013)	0.084		
Original implantation route (reference = IJV)				
SCV	1.622 (0.533,4.933)	0.394		
AxV	0.586 (0.159,2.159)	0.422		
Tumor stage (advanced vs. early)	1.213 (0.413,3.563)	0.725		
Prior ipsilateral radiotherapy (yes vs. no)	0.318 (0.071,1.423)	0.134		
Chemotherapy regimen (combination vs. Single-agent)	0.602 (0.215,1.687)	0.335		

Firth penalized likelihood logistic regression was performed because of the limited number of thrombotic events (n=18). Clinically prespecified covariates were included in the model. Bootstrap resampling with 632 iterations was performed to assess the internal predictive stability of the penalized logistic regression model.

### 3.5 Diagnostic performance of D-dimer for detecting asymptomatic catheter-related thrombosis

ROC analysis demonstrated moderate discrimination of D-dimer for ultrasound-detected catheter-related thrombosis (AUC = 0.721, 95% CI: 0.574–0.868; P = 0.002). The optimal cutoff determined by the Youden index was 0.251 μg/mL. At this threshold, sensitivity was 77.78%, specificity was 76.85%, PPV was 21.88%, NPV was 97.65%, LR+ was 3.36, and LR− was 0.29 (**Figure 3**).

**Figure 3:**
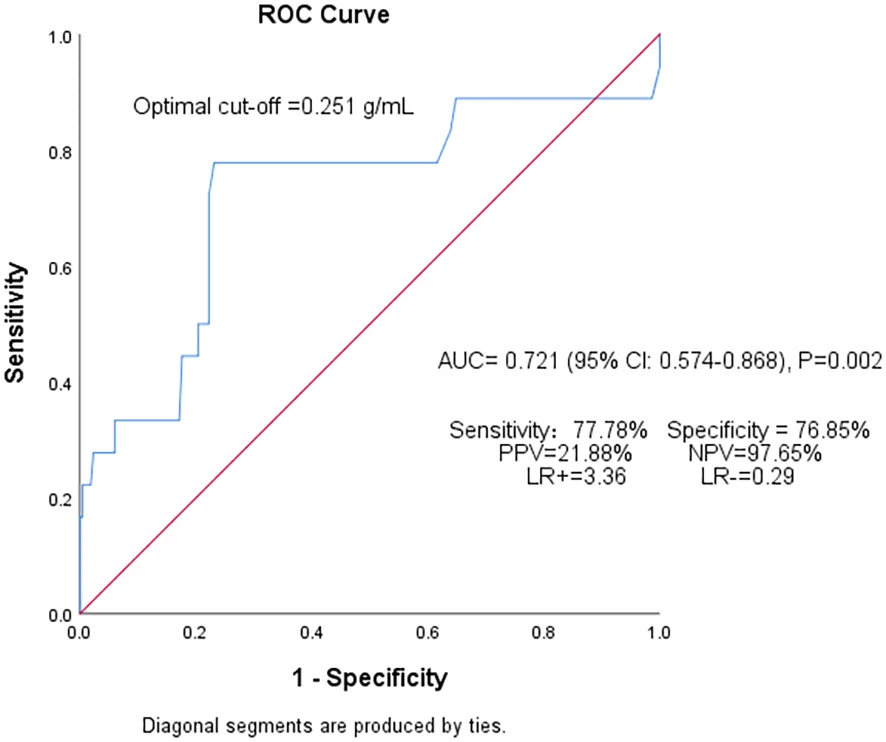
Receiver operating characteristic curve analysis of D-dimer for identifying ultrasound-detected catheter-related thrombosis before TIVAP removal. Receiver operating characteristic (ROC) curve analysis was performed to evaluate the discriminatory ability of pre-removal D-dimer levels for detecting ultrasound-confirmed catheter-related thrombosis. The optimal cutoff value was determined using the Youden index. At the ROC-derived cutoff value of 0.251 μg/mL, D-dimer showed a sensitivity of 77.78%, specificity of 76.85%, positive likelihood ratio (LR+) of 3.36, and negative likelihood ratio (LR−) of 0.29.

### 3.6 Exploratory four-scenario framework based on D-dimer and ultrasonography findings

Based on the ROC-derived D-dimer cutoff of 0.251 μg/mL and ultrasonographic findings, the 234 patients were categorized into four exploratory profiles (**Table 5**; **Figure 4**). Scenario A (D-dimer <0.251 μg/mL and ultrasound negative) included 166 patients (70.9%); Scenario B (D-dimer <0.251 μg/mL and ultrasound positive) included 4 patients (1.7%); Scenario C (D-dimer ≥0.251 μg/mL and ultrasound negative) included 50 patients (21.4%); and Scenario D (D-dimer ≥0.251 μg/mL and ultrasound positive) included 14 patients (6.0%). These categories were intended to describe heterogeneous pre-removal profiles rather than establish validated treatment pathways.

**Table 5:** Exploratory four-scenario framework based on pre-removal D-dimer level and ultrasound findings.

Scenario	D-dimer	Ultrasound finding	Patients n (%)	Exploratory profile
A	<0.251 μg/mL	No thrombosis	166 (70.9)	Double-negative
B	<0.251 μg/mL	Thrombosis present	4 (1.7)	Imaging-positive despite D-dimer below cutoff
C	≥0.251 μg/mL	No thrombosis	50 (21.4)	D-dimer elevation without imaging-confirmed thrombosis
D	≥0.251 μg/mL	Thrombosis present	14 (6.0)	Double-positive

This exploratory classification was generated from observed D-dimer and ultrasound findings and was not designed to establish treatment recommendations.

**Figure 4:**
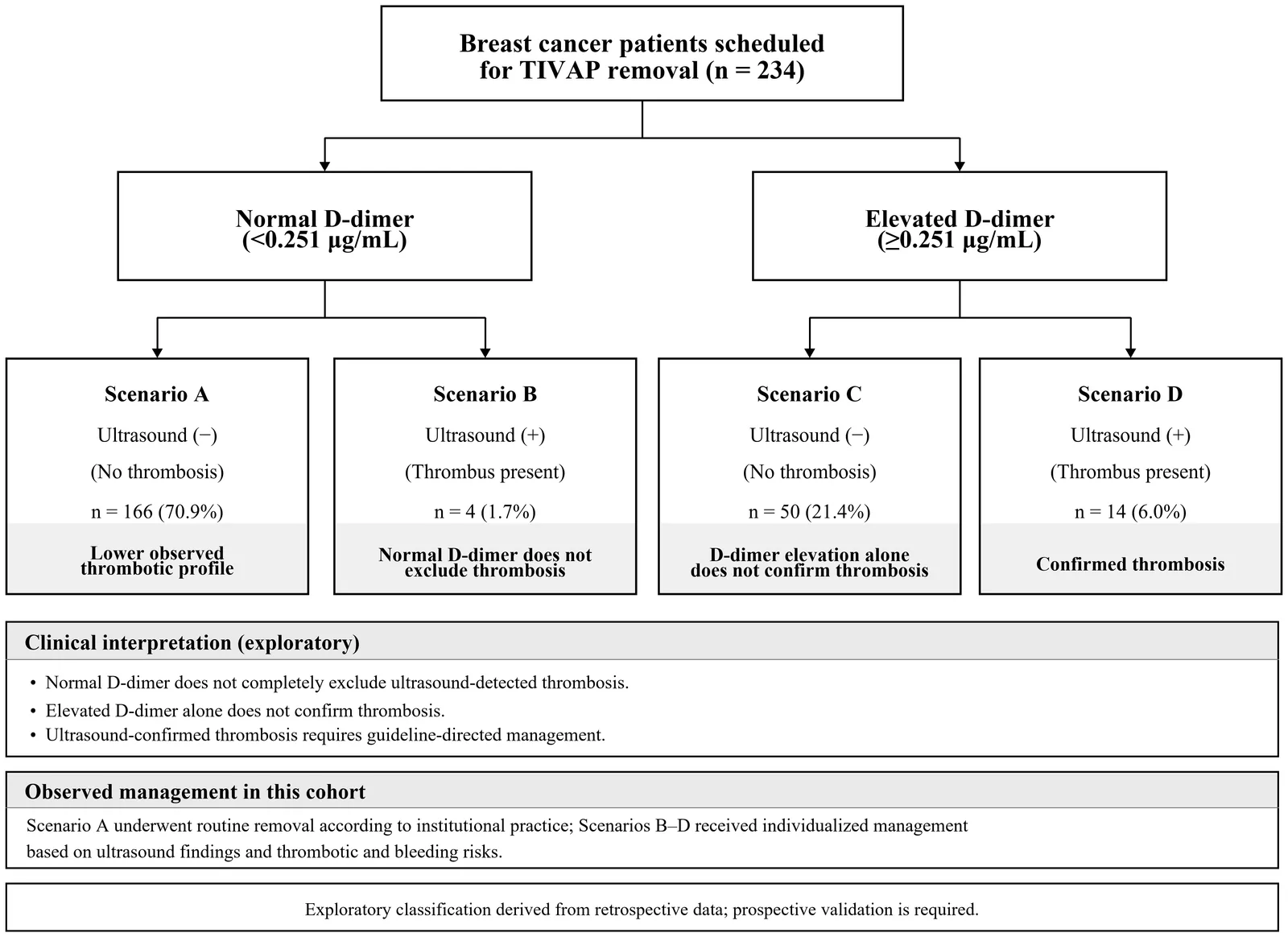
Exploratory pre-removal thrombosis risk-stratification framework based on D-dimer levels and vascular ultrasonography findings. Patients undergoing TIVAP removal were classified according to the combination of pre-removal D-dimer levels and ultrasound findings using the ROC-derived D-dimer cutoff value of 0.251 μg/mL. This framework represents an exploratory classification approach derived from retrospective data integrating biomarker and imaging information before port removal. The clinical implications of these categories and any associated management strategies require validation in prospective studies.

## 4 Discussion

The present study compared the safety outcomes of three commonly used TIVAP implantation routes (IJV, SCV, and AxV) in breast cancer patients and evaluated the utility of pre-removal D-dimer testing combined with vascular ultrasonography for identifying asymptomatic thrombosis before port removal. The findings indicate that the SCV approach was associated with a higher overall complication rate than the IJV and AxV approaches, with catheter fracture and pinch-off syndrome occurring exclusively in the SCV group. In contrast, the AxV approach showed a complication profile comparable to that of the IJV approach. In the exploratory penalized regression analysis, D-dimer level and catheter dwell time were associated with asymptomatic thrombosis; however, effect estimates should be interpreted cautiously given the limited number of events. The combined D-dimer/ultrasonography approach was therefore interpreted as an exploratory classification framework rather than a validated clinical decision model.

### 4.1 Mechanisms underlying safety differences among the three routes

In this study, the SCV approach was associated with a higher incidence of overall complications and severe complications (Clavien–Dindo grade IIIa) compared with the IJV and AxV approaches. However, these findings should be interpreted cautiously because the retrospective design may introduce selection bias and residual confounding, despite the comparable baseline characteristics among groups. Further studies incorporating prospective designs, multivariable adjustment, or propensity score methods are needed to confirm these observations.

The higher complication rate observed with SCV access may be related to anatomical characteristics of the costoclavicular space. Catheters placed through the SCV route pass between the clavicle and the first rib, where repetitive mechanical compression during shoulder movement may result in progressive catheter deformation and eventual fracture. Previous studies have reported a pinch-off syndrome incidence of approximately 1.1%–5.0% among patients with SCV-implanted TIVAPs (18). In the present cohort, the incidence of pinch-off syndrome in the SCV group was 5.8%, which was slightly higher than previously reported. This difference may be associated with the characteristics of the study population, as many breast cancer patients receive chest wall radiotherapy, which may influence local tissue conditions. Regular clinical and imaging surveillance may therefore be particularly important in patients with SCV ports.

Pneumothorax is another potential complication associated with central venous access. A retrospective analysis of 2,897 TIVAP implantations reported a substantially higher pneumothorax rate with SCV puncture than with IJV puncture (4.47% vs. 0.04%) (19). In the present study, pneumothorax occurred in both the SCV and AxV groups (0.7% and 0.6%, respectively), suggesting that AxV access does not completely eliminate this risk. Because the AxV remains anatomically close to the pleural dome, meticulous ultrasound guidance and standardized puncture techniques remain essential for reducing procedure-related complications.

Previous studies have reported procedural advantages of AxV/SCV access, including improved postoperative comfort in a randomized breast cancer trial (14) and shorter operative time with AxV access in a recent three-route comparative cohort (20). In contrast, no significant difference in operative time was observed among the three groups in our study (P = 0.351). Although an operative time advantage for AxV access was not demonstrated, the anatomical advantage of avoiding catheter compression within the costoclavicular space remains a potential benefit. Nevertheless, successful AxV puncture requires adequate ultrasound expertise, and previous studies have reported puncture failure rates of approximately 4.7% (21).

### 4.2 Multifactorial mechanisms of catheter-related thrombosis

In the present study, the original implantation route was not significantly associated with thrombus detection at the time of port removal (P = 0.251). Similarly, operative time did not differ significantly among the three groups (P = 0.351). These findings suggest that, under standardized implantation procedures and continuous ultrasound guidance, the influence of access route on long-term thrombosis risk may be less pronounced than expected.

Anatomical and imaging studies suggest that narrowing of the costoclavicular space and extrinsic compression of the subclavian vein may contribute to venous obstruction and thrombosis (22, 23). However, this association was not confirmed in our cohort, possibly because standardized ultrasound-guided techniques reduced differences related to insertion route. Prospective studies are required to further clarify the relationship between access route and long-term catheter-related thrombosis.

Catheter dwell time was identified as another important factor associated with thrombosis. In the multivariable analysis, longer catheter dwell time was independently associated with an increased risk of asymptomatic thrombosis. In the primary multivariable Firth model, each additional 30 days of catheter dwell time was associated with higher odds of asymptomatic catheter-related thrombosis (adjusted OR = 1.623, 95% CI 1.245–2.114, P<0.001). Prolonged catheter exposure may contribute to endothelial irritation, local inflammation, and thrombus formation. Routine ultrasound screening before port removal in this study may also have facilitated the detection of clinically silent thrombotic events.

Previous meta-analyses have identified catheter malposition, elevated D-dimer levels, increased fibrinogen concentrations, and elevated platelet counts as potential risk factors for catheter-related thrombosis (24). Breast cancer patients may have an underlying hypercoagulable state associated with tumor biology, while systemic anticancer treatments may further modify thrombotic risk (25). In the present study, chemotherapy regimen was evaluated as single-agent versus combination therapy and was not significantly associated with pre-removal thrombosis. However, individual anticancer agents were not analyzed separately. This distinction is clinically relevant because anticancer treatments may differ in their prothrombotic potential. Therefore, CRT is likely the result of multiple interacting factors, including tumor-related hypercoagulability, anticancer treatment, patient characteristics, catheter-related factors, and procedural technique.

The type of central venous access device may also influence thrombotic risk. PICCs are increasingly used in patients with cancer because of their convenience, bedside insertion, and ease of removal; however, several studies have reported a higher risk of catheter-related VTE with PICCs than with PORTs. A recent review by Fioretti et al. summarized evidence suggesting a greater thrombotic burden with PICCs and emphasized the importance of device selection, insertion technique, individualized VTE risk assessment, and ultrasound surveillance (28). PICCs were not included in the present cohort because our predefined objective was to compare three venous access routes for TIVAP implantation rather than different classes of central venous access devices. Future studies directly comparing PICCs and TIVAPs may further clarify how device type interacts with patient-, treatment-, and catheter-related factors to influence thrombosis risk.

### 4.3 Value and limitations of D-dimer in asymptomatic thrombosis screening

In this study, D-dimer showed moderate diagnostic discrimination for asymptomatic thrombosis (AUC = 0.721) but a high negative predictive value (97.65%). These findings suggest that a normal D-dimer level may be useful for identifying patients at relatively low risk of asymptomatic thrombosis. However, the moderate AUC indicates that D-dimer alone cannot provide sufficient accuracy for definitive risk stratification.

Similar findings have been reported by Nañez-Terreros et al. (26), who demonstrated a negative predictive value of 90.9% for peripheral blood D-dimer in asymptomatic catheter-related thrombosis. Notably, 4 of the 18 thrombosis cases (22.2%) had D-dimer levels below the ROC-derived cutoff, indicating that a low D-dimer level alone could not exclude catheter-related thrombosis.

Several mechanisms may explain the presence of thrombosis despite normal D-dimer levels. First, chronic mural thrombi may exhibit limited ongoing fibrin degradation, resulting in lower circulating D-dimer concentrations. Second, small thrombus burden may not sufficiently activate systemic fibrinolysis. Third, the timing of blood sampling may influence D-dimer detection because D-dimer levels fluctuate during thrombus evolution.

In our thrombosis cohort, 16 of 18 patients (88.89%) had mural rather than occlusive thrombi. Mural thrombi generally exhibit lower fibrinolytic activity than acute occlusive thrombi, which may partly explain the occurrence of false-negative D-dimer results. Conversely, some patients without ultrasound-detected thrombus showed relatively elevated D-dimer levels, reflecting the complex hypercoagulable state commonly observed in cancer patients.

### 4.4 Exploratory interpretation of the four-scenario framework

The proposed four-scenario framework integrates pre-removal D-dimer testing and vascular ultrasonography to characterize catheter-related thrombosis status before TIVAP removal. Unlike implantation-related factors, these assessments are performed immediately before device removal and may provide additional information for individualized evaluation. However, this framework should be considered an exploratory classification approach derived from retrospective observations rather than a validated clinical decision model.

The framework highlights that neither D-dimer testing nor ultrasonography alone may fully characterize thrombotic status. Patients with D-dimer levels below the ROC-derived threshold may still have ultrasound-detected thrombosis, whereas isolated D-dimer elevation without imaging evidence of thrombosis should not be interpreted as confirmed venous thromboembolism or an indication for routine anticoagulation. In this cohort, anticoagulation decisions were based on individualized clinical assessment and institutional practice rather than prospective intervention, and current evidence does not support routine pharmacological thromboprophylaxis solely because of catheter presence or isolated D-dimer elevation (27, 28).

Similarly, the anticoagulation and reassessment strategies observed in patients with ultrasound-confirmed thrombosis should not be interpreted as treatment recommendations. The 2–4-week reassessment interval reflected the pre-removal evaluation process in this retrospective cohort rather than the recommended duration of anticoagulant therapy, which should follow guideline-directed management (27).

Because the number of patients in individual scenarios was limited and management decisions were not prospectively assigned, the clinical utility of this framework remains uncertain. Prospective multicenter studies with standardized assessment protocols and predefined management strategies are required to validate whether this exploratory framework can improve pre-removal risk assessment.

### 4.5 Study limitations and future directions

This study has several limitations. First, the retrospective observational design prevented randomization of TIVAP implantation routes. Although baseline characteristics were comparable among groups, residual confounding related to operator preference, venous anatomy, previous treatment history, and institutional practice cannot be excluded. Therefore, differences among implantation routes should be interpreted as associations rather than causal effects. Second, the pre-removal thrombosis analysis included only 234 patients who completed chemotherapy and underwent port removal with available D-dimer testing and ultrasonography. The remaining patients still had indwelling ports or had not reached the removal endpoint during the study period. Although this reflects routine clinical workflow, potential selection bias cannot be completely excluded. In addition, this study focused on asymptomatic catheter-related thrombosis detected by pre-removal ultrasonography, and symptomatic thromboembolic events after port removal could not be systematically evaluated. Third, the relatively small number of thrombotic events limited statistical power. Although Firth penalized logistic regression was applied to reduce small-sample bias, the results require cautious interpretation and external validation. Similarly, the proposed four-scenario framework was derived from retrospective observations and institutional practice patterns; therefore, it should be considered an exploratory pre-removal risk-stratification framework rather than a validated clinical decision model. The anticoagulation approaches observed in this cohort, including the reassessment interval in Scenario D, should not be interpreted as recommendations for treatment duration. Future prospective multicenter studies with standardized pre-removal assessments, detailed anticancer treatment information, longitudinal anticoagulation data, and long-term follow-up are required to validate this exploratory framework and determine its clinical utility.

## 5 Conclusions

In this retrospective cohort, the AxV approach showed a lower observed overall complication rate than the SCV approach, although residual confounding related to non-random route selection cannot be excluded. Pre-removal D-dimer showed moderate discrimination for asymptomatic catheter-related thrombosis and was insufficient as a standalone exclusion test. Integrating D-dimer with vascular ultrasonography allowed patients to be categorized into four exploratory pre-removal profiles. This framework is hypothesis-generating and requires prospective multicenter validation before its clinical utility can be established.

## Data Availability

The original contributions presented in the study are included in the article/supplementary material. Further inquiries can be directed to the corresponding authors.
